# Effects of Malocclusion on Maximal Aerobic Capacity and Athletic Performance in Young Sub-Elite Athletes

**DOI:** 10.3390/sports11030071

**Published:** 2023-03-20

**Authors:** El Mokhtar El Ouali, Hassane Zouhal, Loubna Bahije, Azeddine Ibrahimi, Bahae Benamar, Jihan Kartibou, Ayoub Saeidi, Ismail Laher, Sanae El Harane, Urs Granacher, Abdelhalem Mesfioui

**Affiliations:** 1 Laboratory of Biology and Health, Department of Biology, Ibn Tofail University of Kenitra, Kénitra 14000, Morocco; a.mesfioui@yahoo.fr; 2 Movement, Sport, Health and Sciences Laboratory (M2S), UFR-STAPS, University of Rennes 2-ENS Cachan, Av. Charles Tillon, 35044 Rennes, France; 3 Institut International des Sciences du Sport (2IS), 35850 Irodouer, France; 4 Department of Dentofacial Orthopedics, Faculty of Dental Medicine, Mohammed 5 University of Rabat, Rabat 10000, Morocco; l.bahij@um5s.net.ma; 5 Medical Biotechnology Laboratory, Faculty of Medicine and Pharmacy, Mohamed 5 Rabat University, Rabat 10000, Morocco; a.ibrahimi@um5r.ac.ma; 6 BENAMAR Medical Analysis Laboratory, Rabat 10000, Morocco; bahaelab@hotmail.com; 7 Ministry of National Education and Teaching and Sports, Rabat 10000, Morocco; jihan.kartibou@usmba.ac.ma; 8 Department of Physical Education and Sport Sciences, Faculty of Humanities and Social Sciences, University of Kurdistan, Sanandaj 66177-15175, Iran; saeidi_as68@yahoo.com; 9 Department of Anesthesiology, Pharmacology, and Therapeutics, Faculty of Medicine, University of British Columbia, Vancouver, BC V6T 1Z3, Canada; ismail.laher@ubc.ca; 10 Department of Pathology and Immunology, Faculty of Medicine, University of Geneva, 1211 Geneva, Switzerland; sanae.elharane@unige.ch; 11 Department of Sport and Sport Science, Exercise and Human Movement Science, University of Freiburg, 79102 Freiburg, Germany

**Keywords:** exercise, training, running, orthodontic pathologies, endurance

## Abstract

Oral pathologies can cause athletic underperformance. The aim of this study was to determine the effect of malocclusion on maximal aerobic capacity in young athletes with the same anthropometric data, diet, training mode, and intensity from the same athletics training center. Sub-elite track and field athletes (middle-distance runners) with malocclusion (experimental group (EG); n = 37; 21 girls; age: 15.1 ± 1.5 years) and without malocclusion (control group (CG); n = 13; 5 girls; age: 14.7 ± 1.9 years) volunteered to participate in this study. Participants received an oral diagnosis to examine malocclusion, which was defined as an overlapping of teeth that resulted in impaired contact between the teeth of the mandible and the teeth of the upper jaw. Maximal aerobic capacity was assessed using the VAMEVAL test (calculated MAS and estimated VO_2max_). The test consisted of baseline values that included the following parameters: maximum aerobic speed (MAS), maximal oxygen uptake (VO_2max_), heart rate frequency, systolic (SAP) and diastolic arterial pressure (DAP), blood lactate concentration (LBP), and post-exercise blood lactate assessment (LAP) after the performance of the VAMEVAL test. There were no statistically significant differences between the two study groups related to either anthropometric data (age: EG = 15.1 ± 1.5 vs. CC = 14.7 ± 1.9 years (*p* = 0.46); BMI: EG = 19.25 ± 1.9 vs. CC = 19.42 ± 1.7 kg/m^2^ (*p* = 0.76)) or for the following physical fitness parameters and biomarkers: MAS: EG = 15.5 (14.5–16.5) vs. CG = 15.5 (15–17) km/h (*p* = 0.47); VO_2max_: EG = 54.2 (52.5–58.6) vs. CG = 54.2 (53.4–59.5) mL/kg/min (*p* = 0.62) (IQR (Q1–Q3)); heart rate before the physical test: EG = 77.1 ± 9.9 vs. CG = 74.3 ± 14.0 bpm (*p* = 0.43); SAP: EG = 106.6 ± 13.4 vs. CG = 106.2 ± 14.8 mmHg (*p* = 0.91); DAP: EG = 66.7 ± 9.1 vs. CG = 63.9 ± 10.2 mmHg (*p* = 0.36); LBP: EG = 1.5 ± 0.4 vs. CG = 1.3 ± 0.4 mmol/L (*p* = 0.12); and LAP: EG = 4.5 ± 2.36 vs. CG = 4.06 ± 3.04 mmol/L (*p* = 0.60). Our study suggests that dental malocclusion does not impede maximal aerobic capacity and the athletic performance of young track and field athletes.

## 1. Introduction

Recent advances in sports medicine have allowed athletic performance to be monitored using a variety of assessments, including physical, technical, tactical, physiological, psychological, and medical parameters [1,2]. An athlete’s performance depends on the complex interaction of various physiological systems (e.g., proprioceptive, visual, and vestibular) to enable adequate neuromuscular actions [3,4]. The medical committee of sports clubs and federations primarily monitor performance and physiological measures such as the cardiorespiratory and musculoskeletal system with little attention paid to oral health. However, there is evidence that oral pathologies lead to athletic underperformance, suggesting that the monitoring of oral health should be an integral part of the performance testing of athletes [5].

The maxillary and mandibular teeth interact with each other almost 2000 times a day, mainly during chewing and swallowing [6], but this can also occur before and during physical effort. The normal coordination of dental occlusion is essential during physical activity [6], so that athletes have a greater ability to control balance under different conditions [7] such as the mastery of posture developed by gymnasts and dancers [8,9].

The World Health Organization (WHO) reports that malocclusion is a prevalent oral condition in both children and adults. In fact, it is the second most common disorder in children and the third most common in adults, following cavities and periodontal diseases [10]. Occlusion was defined by Boissonnet in 2011 as “Occlusion is the meshing of teeth so that the teeth of the mandible come into contact with the teeth of the upper jaw” [11]. In other words, if the upper (maxillary) and lower (mandibular) teeth do not make proper contact during closure, it is referred to as malocclusion [12,13,14,15]. More specifically, malocclusion is characterized by a poor alignment between the teeth of the upper and lower dental arches. Dental malocclusion refers to the misalignment of dental arches, which can cause various disturbances. Angle’s classification of occlusion and malocclusion is based on the position of the canines and the first molar in the anteroposterior direction [10]. Children who walk with a proper physiology tend to exhibit regular occlusion and are less likely to experience overloading injuries to their temporomandibular joint (TMJ) or vertebral column. Additionally, these children frequently maintain an appropriate posture [16,17]. Dental occlusion maintains the correct position of the mandible and provides comfort to athletes and/or patients [6]. An untreated malocclusion can affect body balance [18]. A study by Yoshida et al. [19] suggested that a reduced number of remaining natural teeth decreases balance control despite the use of dentures. Periodontal ligaments, masticatory muscles, and ensuring proprioception contribute to the regulation of posture and body movements [20]. The position of the mandible is controlled by the trigeminal nerve, which is influenced by posture [21]. A disorder of dental occlusion stimulates the trigeminal nerve and induces a chain of muscular and articular responses [22].

Maintaining good oral health is crucial for athletes, as oral diseases can have a direct impact on their overall health and prevent them from achieving their full athletic performance [23,24]. Sports dentistry focuses on the critical involvement of dentists in the research, prevention, treatment, rehabilitation, and understanding of the impact of oral diseases on the athletic abilities of professional and amateur athletes whilst improving their performance and reducing the risk of injury [25,26]. Later studies have indicated that dental malocclusion affects posture, contractile muscles, and athletic performance [27]. The peak force in occlusion is significantly higher in professional athletes compared with amateurs. A relationship has been described between the muscles involved in occlusion and the force produced by the postural muscles of the spine [28], so that a dental malocclusion influences the spatial position of the spine [29], body balance [19,20,21], vision [3,30], and eccentric strength of postural muscles [22,31,32,33,34,35,36,37,38]. The impact of dental malocclusion on postural stability and performance has been studied in rifle shooting [38], golf [18], and running [39]. Dental malocclusions can affect the development of the upper jaw, cardiorespiratory efficiency during exercise, and physical capabilities [40]. A large number of high-level athletes wear dental appliances to optimize their dental occlusion, thus maintaining their postural balance and athletic performance [6].

Of the elite athletes who participated in the London 2012 and Rio de Janeiro 2016 Olympic Games, 32% believed their oral health had an impact on their athletic performance and 27% said their oral health had an impact on their overall quality of life [41]. A total of 3% of athletes reported difficulties in participating in training due to oral health problems [26,42] and over 40% of them expressed dissatisfaction with their oral health condition [43]. In Brazil, investigations among footballers, basketball players, and triathletes revealed that 74%, 40%, and 38%, respectively, considered that oral problems interfered with physical performance [44]. Nadia Ouaziz represented Morocco in international 5 km and cross-country competitions in the 1990s. She suffered from chronic neck and back pain that affected her performance and recovery between sessions. A dental malocclusion was diagnosed as the source of her problems and orthodontic treatment with an individualized neuromuscular appliance worn during training sessions and at night produced immediate improvements, as stated by the athlete: “I had a lot more power in my legs. I felt refreshed and recovered between sessions” [40]. In 2009, Aly Cissokho’s transfer to AC Milan football club was cancelled due to the detection of a dental problem by the Italian doctors, which, according to them, could have caused numerous physical problems such as injuries [45]. Over the years 1985–1986, the American sprinter Carl Lewis underwent orthodontic treatment to correct his dental malocclusion and later went on to dominate world sprinting competitions in the 1980s and 1990s [46].

Randomized experimental studies on dental occlusion related to performance are limited, particularly on physical tests and biomarkers evaluating the athletic performance of athletes. According to the different hypotheses above, which suggest a negative impact on one or more parameters of athletic performance [3,6,27,30,40], the objective of our investigation was to examine the impact of malocclusion on the maximal aerobic capacity of young sub-elite athletes from the same training center with great similarity in terms of anthropometry, diet, training (same mode and training load), recovery methods, and medical intervention. We hypothesized that orthodontic pathologies such as malocclusion can negatively influence maximal aerobic capacity.

## 2. Materials and Methods

### 2.1. Study Design

The main objective of the present investigation was to assess the impact of malocclusion on the physical and physiological capacities of young athletes. We chose a very homogeneous and identical population of athletes (young athletes living in the same training center with the same diet, lifestyle, athletic specialty, training, and medical follow-up) in order to show whether a difference in athletic performance between the control group and the experimental group would be due only to malocclusion.

### 2.2. Inclusion and Exclusion Criteria of Participants

Participants were recruited according to the following inclusion criteria: young sub-elite middle-distance runners under 18 years of age from the same training center with the same anthropometric data (difference not significant), nutrition, load training, and recovery techniques. The exclusion criteria were a history of musculoskeletal injuries and disorders, significant difference in anthropometric data, smoking, and consumption of alcohol or the regular use of medication for any reason. The participants were required to avoid any physical activity for 48 h before the day of the test and were excluded from the study if they were uncomfortable with any aspect of the experimental protocol.

### 2.3. Procedures

Written parental informed consent was obtained from all subjects. This study was approved by the Ethics Committee of the Doctoral Centre for Human Biomedical Research at the Faculty of Science, Ibn Tofail University (Kenitra, Morocco) and met the requirements for conducting biomedical research involving human subjects.

**Figure 1:**
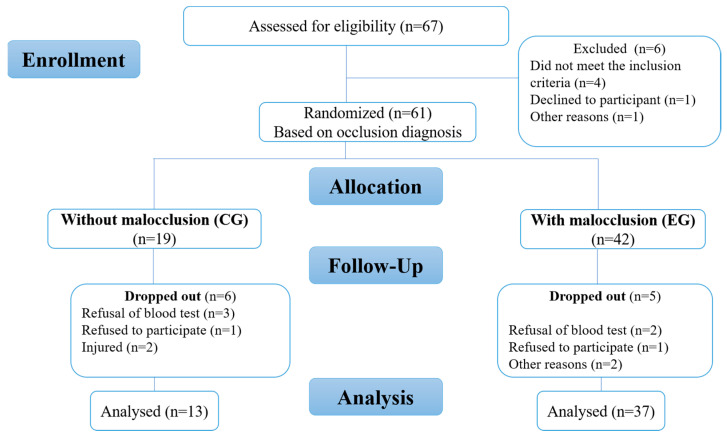
Flow diagram of study.

A total of 50 young (n = 50; age range: 12–17 years old) highly trained/national-level athletes according to a 6-tiered Participant Classification Framework developed by Alannah et al. [47] participated in the study. A CONSORT flow diagram was included (Figure 1). All subjects were from the Athletics Training Center in Rabat (Center of the Royal Armed Forces, Morocco) and had the same diet and followed regular training programs of the same intensity (five times a week) and were followed by the same medical staff using the same recovery techniques (emersion in cold water and massage). The participants were affiliated with the Royal Moroccan Athletics Federation and competed in middle-distance races at the National Championship level. Orthodontic specialists from the Rabat Dental Consultation and Treatment Centre initially assessed the occlusal status of the athletes using Angle’s molar classification, and were classified as class I (no malocclusion, but crowding or misalignment of teeth), class II (distoocclusions) divisions 1 and 2, and class III (mesiocclusion) [10,46]. The maximum aerobic capacity was measured by baseline values of the following parameters: maximal aerobic speed (MAS), VO_2max_, heart rate, systolic blood pressure (SAP), diastolic blood pressure (DAP), blood lactate concentration (LBP), and post-exercise blood lactate concentration (LAP).

### 2.4. Maximal Aerobic Capacity

We used the VAMEVAL test to evaluate the MAS [48]. This test assesses the MAS to estimate VO_2max_ by running at a progressively faster pace in one minute increments. The rhythm was stored as an MP3 file containing sound signals.

The physical test took place on a track that met the standards of the International Athletics Federation. Runners proceeded to blocks that were spaced every 20 m when prompted by the sound of a beep. The speed was then gradually increased by 0.5 km/h every minute. Participants stopped when they were no longer able to maintain the imposed rhythm, at which point the MAS and VO_2max_ were evaluated using a reference table.

### 2.5. Blood Pressure and Heart Rate Measurements

To obtain the baseline values of systolic and diastolic blood pressure and heart rate, measurements were only taken after the athletes rested for 15 min on chairs, using digital arm blood pressure monitors (Bosch, Sohn, Germany). The recorded values were the average of three measurements. Our measurement method respected and adhered to the recommendations for blood pressure measurements [49,50].

### 2.6. Blood Lactate Measurements

With the help of the health staff and following the safety recommendations, we sampled 2 mL of blood from our athletes from 9 am to 10 am. Blood lactate concentrations were measured at rest and 3 min after the VAMEVAL physical fitness test. A total of 50 tubes containing heparin were used to store 3 mL blood samples, which were then centrifuged (Hettich^®^ ROTOFIX 32A, Tuttlingen, Germany) for five minutes at 3000 revolutions/min. The plasma was then separated by micropipettes and placed in Eppendorf tubes. The Eppendorf tubes were then placed in coolers and taken to the laboratory for the analysis. Lactate levels were measured using an enzymatic colorimetric method (Roche Diagnostics Cobas C311, Creatinine Jaffe Gen.2, Singapore) [51].

### 2.7. Statistical Analyses

To assess the normality of the data, we used the visual method (QQ-Plot) and the Agostino–Pearson statistical test. The heart rate, SAP, DAP, LBP, and LAP were normally distributed whereas the MAS and VO_2max_ were not normally distributed. For the normally distributed baseline data, we used a *t*-test (parametric test); a Mann–Whitney test (non-parametric test) was used for the non-normally distributed baseline data. To analyze the differences in blood lactate before and after physical effort in both groups, we conducted a mixed-effects analysis for repeated measures analysis of variance (ANOVA), and subsequently performed Tukey’s multiple comparison test. Accordingly, the data were presented as means and standard deviations (SD), t-statistic (t), and degrees of freedom (df) for the normally distributed data or as medians and interquartile ranges (IQR (Q1–Q3)) for the non-normally distributed data. In addition, the comparison of the baseline and pre–post data (blood lactate) between the EG and CG was displayed with 95% confidence intervals of the difference (95% CI). The statistical significance was set at *p* < 0.05 for all analyses. The statistical analysis was performed with GraphPad Prism 9.2.0 statistical software (GraphPad Software Inc., San Diego, CA, USA).

## 3. Results

**Figure 2:**
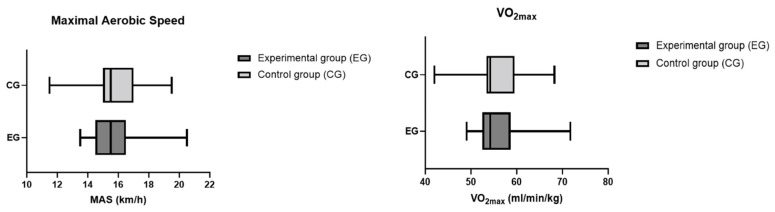
MAS and VO_2max_ values in the control and experimental groups using Mann–Whitney test. Values are presented as median and IQR (Q1–Q3). Differences between the two groups were not statistically significant (*p* > 0.05).

**Figure 3:**
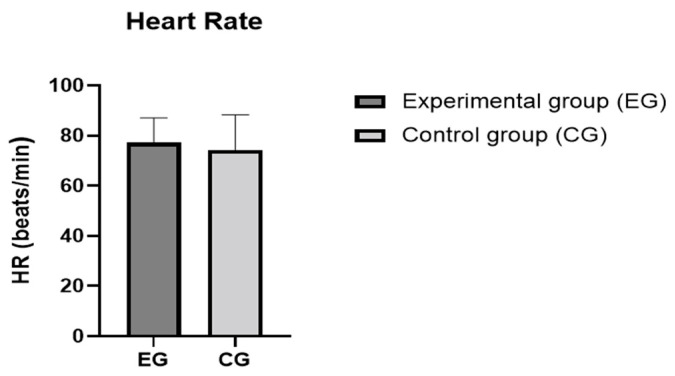
Heart rates of the control and experimental groups. The values are presented as means and SD. Differences between the two groups were not statistically significant (*p* > 0.05).

**Figure 4:**
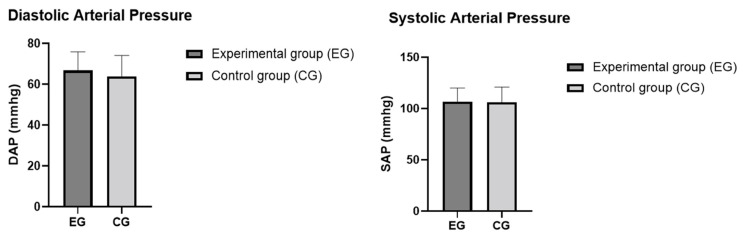
SAP and DAP in the control and experimental groups. The values are presented as means and SD. Differences between the two groups were not statistically significant (*p* > 0.05).

**Figure 5:**
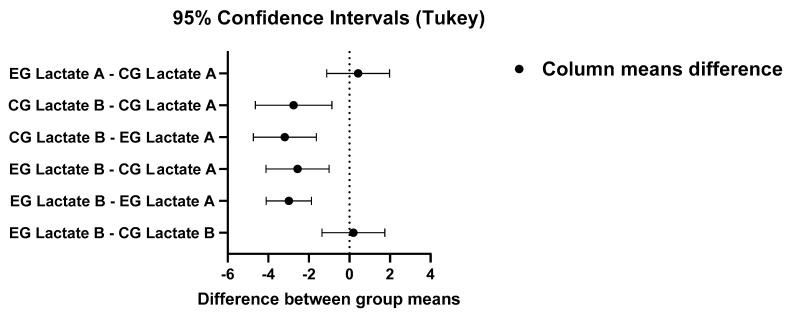
Tukey’s multiple comparison test of blood lactate before and after physical effort between EG and CG (one-way ANOVA).

A total of 50 young athletes volunteered to participate in this study. After a diagnosis of malocclusion, 13 athletes (5 females and 8 males) without malocclusion formed the control group (CG) and 37 athletes (21 females and 16 males) with malocclusion formed the experimental group (EG). The anthropometric data in the control and experimental groups were similar (age: *p* = 0.46; BMI: *p* = 0.76), as shown in Table 1. The values for the MAS, VO_2max_, heart rate, SAP, DAP, LBP, and LAP in the control and experimental groups are summarized in Table 2 and Table 3. After analyzing our data, we found that the differences in the athletic performance and biomarkers were not statistically significant between the athletes with malocclusions and those without malocclusions, as summarized in Figure 2, Figure 3, Figure 4 and Figure 5. The values were the mean ± SD for the normally distributed parameters (heart rate, SAP, DAP, LBP, and LAP) and the median and IQR (Q1–Q3) for the non-normally distributed parameters (MAS and VO_2max_), with a 95% confidence interval difference (95% CI) for the comparison of data in the EG and CG during the baseline and pre–post measurements (blood lactate; Table 4).

## 4. Discussion

Studies examining the association between malocclusion and athletic performance based on several parameters and biomarkers (physical, physiological, and biomechanical) are relatively limited. The aim of this study was to assess the impact of malocclusion on physical and physiological abilities in an identical population of sub-elite runners from the same training center. The results of our study indicated that the MAS, VO_2max_, heart rate, SAP, DAP, LBP, and LAP were not significantly different (*p* > 0.05) in elite athletes with good dental occlusion compared with those with malocclusion. However, the findings of this investigation were not in agreement with several studies. Eberhard et al. [52] showed that VO_2max_ levels were impaired in subjects with mild, moderate, and severe periodontitis. Athletic performance was demonstrated to be related to a healthy occlusal balance, which is required for optimal postural balance, injury prevention, and also improved muscle strength [30,53]. Mouth breathing and temporomandibular dysfunction were influenced by malocclusions [46]. Dental trauma had a negative impact on athletic performance, which could be remediated by orthodontic treatment [18]. Poor oral health reduced the physical and physiological capacity of professional soccer players [43,54], and dental malocclusion was associated with a loss of muscle strength in the elderly [55]. There were significant alterations in muscle strength after the disturbance of dental occlusion in healthy women [37], and increases in the muscle activity of the masseter, anterior, temporal, and trapezius muscles occurred in professional ballet dancers six months after gnathological treatment [56]. The oral health of athletes and/or individuals can be improved by the application of an occlusal splint [57]. In accordance with our results, Parrini et al. [58] showed that in athletes with malocclusion, no statistically significant differences were observed between the untreated control group and the treated group when performing countermovement and drop jumps or 10 m and 30 m sprint tests. A recent study reported that changes in dental occlusion did not affect the body posture and muscle activity of the upper limbs in male shooters [59].

The heart rate is regulated by the sympathetic and parasympathetic nervous systems. A consideration of heart rate variability (HRV) (the variation between two consecutive heartbeats, or “R–R”) is useful in controlling the training load, monitoring recovery kinetics, and assessing the training condition of athletes. HRV is influenced by temporomandibular joint dysfunction [60] and malocclusion [61]. Orthodontic treatment leads to improvements in oral function and also in HRV [62]. Ekuni et al. [60] found that the heart rate was higher in young adults with malocclusion.

Normal concentrations of blood lactate are 1 to 2 mmol/L at rest, but reach higher values during intense exercise; for example, average values of between 4 and 8 mmol/L have been recorded in soccer matches [63,64]. The blood lactate level is a biochemical marker of muscle fatigue. According to Durst et al. [65], the blood lactate concentration and heart rate are the best biomarkers of the internal load during physical effort. The lactate levels were similar in both groups in our study, and no association was noted between malocclusion and blood lactate levels. To our knowledge, no study has examined changes in lactate levels in relation to dental malocclusion.

A recent study reported that occlusal disturbances negatively influenced athletic performance, where there were increases in athletes with asymmetric muscle contractions (*p* = 0.025) coupled with decreased muscle power (*p* = 0.030) [18]. A significant influence of dental/facial trauma on physical performance (*p* = 0.006) in young professional volleyball and soccer athletes has also been reported [66]. Malocclusion and its treatments can influence body posture, foot–ground contact, center of mass, footprint, etc. [67,68]. Orthodontic treatment involves aligning or moving teeth to improve their appearance and function. Several studies have suggested that orthodontic treatment has a significant positive impact on the technical correction of malocclusion [69,70,71,72,73,74]. Hard stabilization splints (HSS) are utilized to ease tension in the masticatory muscles whilst directing the mandible to a stable position; these splints boast a simple preparation and easy adaptation, making them effective in reducing clinical symptoms [75,76]. In certain cases, prior to orthodontic treatment, therapeutic interventions such as HSS, counseling, or specific exercises may be employed to alleviate TMJ symptoms and minimize the associated discomfort [77,78]. After isokinetic testing, an increase in quadriceps muscle strength was observed when patients wore occlusal treatment splints [7].

Several factors such as genetics and the environment or a combination of both may be responsible for the high prevalence rates of malocclusion [79,80]. *ACTN3* is a gene that codes for an α-actinin-3 protein, a cytoskeletal protein that binds to actin filaments and crosslinks them into dense bodies at the Z-disc of the sarcomere to maintain the myofibrillar network during muscle contraction [81], and is only expressed in type 2 muscle fibers that undergo rapid glycolysis and oxidation [81,82]. A *R577X* polymorphism (rs1815739) of the *ACTN3* gene results from the replacement of arginine by a premature stop codon [83]. The expression of the *ACTN3* (RR) genotype is higher in elite power/sprint athletes than in healthy non-athletes [84]. A low expression of the α-actinin-3 protein occurs in individuals of genotype (XX); on the other hand, in homozygous individuals, the genotype (RR) and/or heterozygotes (RX) have a higher expression of α-actinin-3 [85]. The absence of α-actinin-3 protein expression does not lead to any pathology, but can reduce muscle strength and cause intolerance to physical effort, with a dominance of explosive muscle actions [86] and lower bone mineral density [87]. A study by Zebrick et al. [81] reported a strong correlation between the *ACTN3* 577 (XX) genotype and skeletal class 2 malocclusion (*p* < 0.01) and significantly smaller type 2 fast-fiber diameters in masseter muscles in genotype (XX) subjects (*p* = 0.002). In a similar context, Cunha et al. [88] evaluated two genetic variants of the *ACTN3* gene (rs1518739 and rs678397) with malocclusion. Concerning the rs1518739 variation, they found a significant association between genotype (XX) and skeletal class 2 malocclusion (*p* < 0.05); for the rs678397 variation, they observed a significant association with malocclusion (*p* < 0.05). Our results indicated that malocclusion does not negatively affect the physical and physiological markers of performance in young athletes.

## 5. Limitations

To our knowledge, our study is the first to examine the relationship between malocclusion and the maximal aerobic capacity of athletes, based on a study of a limited number of biomarkers of sports performance. There are a few limitations to our study, which are: (i) the relatively small number of study participants (n = 50), which may be related to an invasive component of the study that may have triggered a degree of anxiety in adolescents; and (ii) the imbalance in the number of athletes without malocclusion (n = 13) compared with the athletes with malocclusion (n = 37). According to Bichara et al. [89], the average treatment time for malocclusions was 30.27 months; however, a comparative study assessing the impact of malocclusion on athletic performance before and after orthodontic treatment follow-up would be very relevant. The follow-up of a large number of athletes undergoing orthodontic treatment and the assessment of several performance parameters and biomarkers before and after treatment would be very useful to answer the question of the association between malocclusion and athletic performance.

## 6. Conclusions

Our study indicated that malocclusion does not negatively affect the maximal aerobic capacity of young sub-elite athletes. Other studies have indicated a significant negative influence of malocclusions on athletic performance. These contradictory findings suggest that further studies are needed.

## Figures and Tables

**Table 1:** Anthropometric data of experimental (EG, with malocclusion) and control (CG, no malocclusion) groups. Values are presented as means, SD, and 95% CI.

Anthropometric Data	With Malocclusion (Mean ± SD)	Without Malocclusion (Mean ± SD)	95% CI of Difference	*p*-Value (*t*-Test)
Age (years)	15.1 ± 1.5	14.7 ± 1.9	(−1.43, 0.65)	0.46
Body height (cm)	170 ± 10	177 ± 12	(−0.004, 0.14)	0.06
Body Mass (kg)	62.5 ± 9.9	69.5 ± 10.9	(0.17, 13.8)	0.04
BMI (kg/m^2^)	19.25 ± 1.9	19.42 ± 1.7	(−1.05, 1.42)	0.76

SD: standard deviation; BMI: body mass index; 95% CI: confidence interval.

**Table 2:** Biological parameters (not normally distributed) in the experimental and control groups. Values are presented as median and IQR (Q1–Q3) and 95% CI.

Biological Parameters (Not Normally Distributed)	With Malocclusion Median (IQR (Q1–Q3))	Without Malocclusion Median (IQR (Q1–Q3))	95% CI of Difference	*p*-Value (Mann–Whitney Test)
Maximal aerobic speed (km/h)	15.5 (14.5–16.5)	15.5 (15–17)	(−1.00, 1.00)	0.47
VO_2max_ (mL/min/kg)	54.2 (52.5–58.6)	54.2 (53.4–59.5)	(−3.50, 3.50)	0.62

**Table 3:** Biological parameters (normally distributed) in the experimental and control groups. Values are presented as means, SD, 95% CI, t-statistic (t), and degrees of freedom (df).

Biological Parameters (Normally Distributed)	With Malocclusion (Mean ± SD)	Without Malocclusion (Mean ± SD)	t, df	95% CI of Difference	*p*-Value (*t*-Test)
Resting heart rate (beats/min)	77.1 ± 9.9	74.3 ± 14.0	0.80, 48	(−10.01, 4.35)	0.43
Systolic arterial pressure (mmHg)	106.6 ± 13.4	106.2 ± 14.8	0.10, 48	(−9.42, 8.5)	0.91
Diastolic arterial pressure (mmHg)	66.7 ± 9.1	63.9 ± 10.2	0.91, 48	(−8.9, 3.32)	0.36
Lactate (before physical effort) (mmol/L)	1.5 ± 0.4	1.3 ± 0.4	1.56, 48	(−0.45, 0.06)	0.12
Lactate (after physical effort) (mmol/L)	4.5 ± 2.36	4.06 ± 3.04	0.51, 48	(−2.10, 1.24)	0.60

SD: standard deviation; 95% CI: confidence interval; t: t-statistic; df: degrees of freedom.

**Table 4:** Comparison of blood lactate before and after physical effort between EG and CG (Tukey’s multiple comparison test). Data are presented as mean difference and 95% CI difference.

Tukey’s Multiple Comparison Test	Mean Difference	95% CI of Difference	Significant	Adjusted *p*-Value
EG Lactate B vs. CG Lactate B	0.2	(−1.35, 1.75)	ns	0.98
EG Lactate B vs. EG Lactate A	−2.10	(−4.10, −1.87)	****	<0.0001
EG Lactate B vs. CG Lactate A	−2.55	(−4.10, −1.005)	***	0.0002
CG Lactate B vs. EG Lactate A	−3.18	(−4.73, −1.63)	****	<0.0001
CG Lactate B vs. CG Lactate A	−2.75	(−4.64, −0.86)	**	0.001
EG Lactate A vs. CG Lactate A	0.43	(−1.12, 1.10)	ns	0.88

A: after; B: before; 95% CI: confidence interval. The level of significance was *p* < 0.05 for all comparisons; ns: non-significant; **: significant *p* < 0.01; ***: significant *p* < 0.001; ****: significant *p* < 0.0001.

## Data Availability

The datasets generated and/or analyzed during the current study are not publicly available. Upon request, the corresponding author will share the dataset.

## References

[1] Desgorces F.-D., Berthelot G., Helou N.E., Thibault V., Guillaume M., Tafflet M., Hermine O., Toussaint J.-F. (2008). From Oxford to Hawaii Ecophysiological Barriers Limit Human Progression in Ten Sport Monuments. PLoS ONE.

[2] Williams A.G., Folland J.P. (2008). Similarity of Polygenic Profiles Limits the Potential for Elite Human Physical Performance. J. Physiol..

[3] Isableu B., Ohlmann T., Cremieux J., Amblard B. (1997). Selection of Spatial Frame of Reference and Postural Control Variability. Exp. Brain Res..

[4] Johnston R.B., Howard M.E., Cawley P.W., Losse G.M. (1998). Effect of Lower Extremity Muscular Fatigue on Motor Control Performance. Med. Sci. Sport Exerc..

[5] Lamendin H., Guendouz B. (1990). Sante Bucco-Dentaire et Performances Sportives. Médecine D’afrique Noire.

[6] Picart P. (2015). Occlusion Dentaire, Posture et Performances Sportives. Ph.D. Thesis.

[7] Baldini A., Beraldi A., Nota A., Danelon F., Ballanti F., Longoni S. (2012). Gnathological Postural Treatment in a Professional Basketball Player: A Case Report and an Overview of the Role of Dental Occlusion on Performance. Ann. Stomatol..

[8] Golomer E., Crémieux J., Dupui P., Isableu B., Ohlmann T. (1999). Visual Contribution to Self-Induced Body Sway Frequencies and Visual Perception of Male Professional Dancers. Neurosci. Lett..

[9] Vuillerme N., Danion F., Forestier N., Nougier V. (2002). Postural Sway under Muscle Vibration and Muscle Fatigue in Humans. Neurosci. Lett..

[10] Cabrera-Domínguez M.E., Domínguez-Reyes A., Pabón-Carrasco M., Pérez-Belloso A.J., Coheña-Jiménez M., Galán-González A.F. (2021). Dental Malocclusion and Its Relation to the Podal System. Front. Pediatr..

[11] Sport et Santé Bucco-Dentaire—2011. https://docplayer.fr/9579536-Sport-et-sante-bucco-dentaire.html.

[12] Asgari I., Soltani S., Sadeghi S.M. (2020). Effects of Iron Products on Decay, Tooth Microhardness, and Dental Discoloration: A Systematic Review. Arch. Pharm. Pract..

[13] Alamri A.M., Alshammery H.M., Almughamis M.A., Alissa A.S., Almadhi W.H., Alsharif A.M. (2019). Dental Recession Aetiology, Classification and Management. Arch. Pharm. Pract..

[14] Kharalampos M. (2020). Comprehensive Patient Rehabilitation While Performing Immediate Dental Implant Placement with the Use of Information-Wave Therapy (Literature Overview). J. Adv. Pharm. Educ. Res..

[15] Bulgakova A.I., Vasilyeva N.A., Vasilyev E.A. (2019). The Clinical and Immunological Rationale for the Use of Prolonged Action Dental Ointment in Periodontology. J. Adv. Pharm. Educ. Res..

[16] Rodríguez S.G., Rodríguez M.L., Ramos L.P. (2017). Modifications of the dental occlusion and its relation with the body posture in Orthodontics. Bibliographic review. Rev. Habanera De Cienc. Médicas.

[17] Scharnweber B., Adjami F., Schuster G., Kopp S., Natrup J., Erbe C., Ohlendorf D. (2017). Influence of Dental Occlusion on Postural Control and Plantar Pressure Distribution. Cranio.

[18] Leroux E., Leroux S., Maton F., Ravalec X., Sorel O. (2018). Influence of Dental Occlusion on the Athletic Performance of Young Elite Rowers: A Pilot Study. Clinics.

[19] Yoshida M., Kikutani T., Okada G., Kawamura T., Kimura M., Akagawa Y. (2009). The Effect of Tooth Loss on Body Balance Control among Community-Dwelling Elderly Persons. Int. J. Prosthodont..

[20] Hoppe C.B., Oliveira J.A.P., Grecca F.S., Haas A.N., Gomes M.S. (2017). Association between Chronic Oral Inflammatory Burden and Physical Fitness in Males: A Cross-Sectional Observational Study. Int. Endod. J..

[21] Sakaguchi K., Mehta N.R., Abdallah E.F., Forgione A.G., Hirayama H., Kawasaki T., Yokoyama A. (2007). Examination of the Relationship between Mandibular Position and Body Posture. Cranio.

[22] Clauzade M. (2007). Orthoposturodontie. Actual. Odonto-Stomatol..

[23] Alves D.C.B., Anjos V.D.L.D., Giovannini J.F.B.G., Lima R.P.E., Mendonça S.M.S. (2017). Odontologia no Esporte: Conhecimento e Hábitos de Atletas do Futebol e Basquetebol Sobre Saúde Bucal. Rev. Bras. Med. Esporte.

[24] D’Ercole S., Tieri M., Martinelli D., Tripodi D. (2016). The Effect of Swimming on Oral Health Status: Competitive versus Non-Competitive Athletes. J. Appl. Oral Sci..

[25] Ashley P., Iorio A.D., Cole E., Tanday A., Needleman I. (2015). Oral Health of Elite Athletes and Association with Performance: A Systematic Review. Br. J. Sports Med..

[26] Gallagher J., Ashley P., Petrie A., Needleman I. (2018). Oral Health and Performance Impacts in Elite and Professional Athletes. Community Dent. Oral Epidemiol..

[27] Moon H.-J., Lee Y.-K. (2011). The Relationship Between Dental Occlusion/Temporomandibular Joint Status and General Body Health: Part 1. Dental Occlusion and TMJ Status Exert an Influence on General Body Health. J. Altern. Complement. Med..

[28] Iwasaki H., Inaba R., Iwata H. (1994). Biting force and physical fitness in athletes. Nihon Eiseigaku Zasshi.

[29] Ohlendorf D., Seebach K., Hoerzer S., Nigg S., Kopp S. (2014). The Effects of a Temporarily Manipulated Dental Occlusion on the Position of the Spine: A Comparison during Standing and Walking. Spine J..

[30] Jakush J. (1982). Divergent Views: Can Dental Therapy Enhance Athletic Performance?. J. Am. Dent. Assoc..

[31] Sforza C., Tartaglia G.M., Solimene U., Morgun V., Kaspranskiy R.R., Ferrario V.F. (2006). Occlusion, Sternocleidomastoid Muscle Activity, and Body Sway: A Pilot Study in Male Astronauts. CRANIO^®^.

[32] Abduljabbar T., Mehta N.R., Forgione A.G., Clark R.E., Kronman J.H., Munsat T.L., George P. (1997). Effect of Increased Maxillo-Mandibular Relationship on Isometric Strength in TMD Patients with Loss of Vertical Dimension of Occlusion. CRANIO^®^.

[33] AL-Abbasi H., Mehta N.R., Forgione A.G., Clark R.E. (1999). The Effect of Vertical Dimension and Mandibular Position on Isometric Strength of the Cervical Flexors. CRANIO^®^.

[34] Santander H., Miralles R., Jimenez A., Zuñiga C., Rocabado M., Moya H. (1994). Influence of Stabilization Occlusal Splint on Craniocervical Relationships. Part II: Electromyographic Analysis. CRANIO^®^.

[35] Ceneviz C., Mehta N.R., Forgione A., Sands M.J., Abdallah E.F., Lobo Lobo S., Mavroudi S. (2006). The Immediate Effect of Changing Mandibular Position on the EMG Activity of the Masseter, Temporalis, Sternocleidomastoid, and Trapezius Muscles. CRANIO^®^.

[36] Takada Y., Miyahara T., Tanaka T., Ohyama T., Nakamura Y. (2000). Modulation of H Reflex of Pretibial Muscles and Reciprocal Ia Inhibition of Soleus Muscle During Voluntary Teeth Clenching in Humans. J. Neurophysiol..

[37] Grosdent S., O’Thanh R., Domken O., Lamy M., Croisier J.-L. (2014). Dental Occlusion Influences Knee Muscular Performances in Asymptomatic Females. J. Strength Cond. Res..

[38] Gangloff P., Louis J.P., Perrin P.P. (2000). Dental Occlusion Modifies Gaze and Posture Stabilization in Human Subjects. Neurosci. Lett..

[39] Maurer C., Stief F., Jonas A., Kovac A., Groneberg D.A., Meurer A., Ohlendorf D. (2015). Influence of the Lower Jaw Position on the Running Pattern. PLoS ONE.

[40] Budd S.C., Egea J.-C. (2017). Dental Occlusion and Athletic Performance. Sport and Oral Health: A Concise Guide.

[41] De Souza J.J., Grande R.S., Bahls R., Santos F.A. (2020). Evaluation of the Oral Health Conditions of Volleyball Athletes. Rev. Bras. Med. Esporte.

[42] Kragt L., Moen M.H., Van Den Hoogenband C.-R., Wolvius E.B. (2019). Oral Health among Dutch Elite Athletes Prior to Rio 2016. Phys. Sport.

[43] Needleman I., Ashley P., Petrie A., Fortune F., Turner W., Jones J., Niggli J., Engebretsen L., Budgett R., Donos N. (2013). Oral Health and Impact on Performance of Athletes Participating in the London 2012 Olympic Games: A Cross-Sectional Study. Br. J. Sports Med..

[44] Do Nascimento B.L., Zen I.R., Demenech L.S., de Oliveira Mazzetto N.C., Spada P.C.P. (2015). Knowledge of Triathlon Athletes about the Relationship between Oral Health and Performance. RSBO.

[45] Manson J. (2010). Influence de L’occlusion sur les Performances Sportives. Ph.D. Thesis.

[46] De Souza L.A., Elmadjian T.R., Brito e Dias R., Coto N.P. (2011). Prevalence of Malocclusions in the 13–20-Year-Old Categories of Football Athletes. Braz. Oral Res..

[47] McKay A.K.A., Stellingwerff T., Smith E.S., Martin D.T., Mujika I., Goosey-Tolfrey V.L., Sheppard J., Burke L.M. (2022). Defining Training and Performance Caliber: A Participant Classification Framework. Int. J. Sport. Physiol. Perform..

[48] Cazorla G. (2014). 3. Evaluation des capacités aérobies. Evaluation des Capacites Physiologiques et Physiques.

[49] Stergiou G.S., Palatini P., Asmar R., Ioannidis J.P., Kollias A., Lacy P., McManus R.J., Myers M.G., Parati G., Shennan A. (2019). Recommendations and Practical Guidance for Performing and Reporting Validation Studies According to the Universal Standard for the Validation of Blood Pressure Measuring Devices by the Association for the Advancement of Medical Instrumentation/European Society of Hypertension/International Organization for Standardization (AAMI/ESH/ISO). J. Hypertens..

[50] Wang J.-G., Bu P.-L., Chen L.-Y., Chen X., Chen Y.-Y., Cheng W.-L., Chu S.-L., Cui Z.-Q., Dai Q.-Y., Feng Y.-Q. (2020). 2019 Chinese Hypertension League Guidelines on Home Blood Pressure Monitoring. J. Clin. Hypertens..

[51] Gar C., Rottenkolber M., Haenelt M., Potzel A.L., Kern-Matschilles S., Then C., Seissler J., Bidlingmaier M., Lechner A. (2020). Altered Metabolic and Hormonal Responses to Moderate Exercise in Overweight/Obesity. Metabolism.

[52] Eberhard J., Stiesch M., Kerling A., Bara C., Eulert C., Hilfiker-Kleiner D., Hilfiker A., Budde E., Bauersachs J., Kück M. (2014). Moderate and Severe Periodontitis Are Independent Risk Factors Associated with Low Cardiorespiratory Fitness in Sedentary Non-Smoking Men Aged between 45 and 65 Years. J. Clin. Periodontol..

[53] Gelb H., Mehta N.R., Forgione A.G. (1995). Relationship of Muscular Strength to Jaw Posture in Sports Dentistry. N. Y. State Dent. J..

[54] Gay-Escoda C., Vieira-Duarte-Pereira D.-M., Ardèvol J., Pruna R., Fernandez J., Valmaseda-Castellón E. (2011). Study of the Effect of Oral Health on Physical Condition of Professional Soccer Players of the Football Club Barcelona. Med. Oral Patol. Oral Cir. Bucal.

[55] Okuyama N., Yamaga T., Yoshihara A., Nohno K., Yoshitake Y., Kimura Y., Shimada M., Nakagawa N., Nishimuta M., Ohashi M. (2011). Influence of Dental Occlusion on Physical Fitness Decline in a Healthy Japanese Elderly Population. Arch. Gerontol. Geriatr..

[56] Didier H., Assandri F., Gaffuri F., Cavagnetto D., Abate A., Villanova M., Maiorana C. (2021). The Role of Dental Occlusion and Neuromuscular Behavior in Professional Ballet Dancers’ Performance: A Pilot Study. Healthcare.

[57] Cesanelli L., Cesaretti G., Ylaitė B., Iovane A., Bianco A., Messina G. (2021). Occlusal Splints and Exercise Performance: A Systematic Review of Current Evidence. Int. J. Environ. Res. Public Health.

[58] Parrini S., Rossini G., Nebiolo B., Airale M., Franceschi A., Cugliari G., Deregibus A., Castroflorio T. (2022). Variations in Athletic Performance with Occlusal Splint in Track and Field Athletes: A Randomized Clinical Trial. J. Sport. Med. Phys. Fit..

[59] Dias A.A., Redinha L.A., Silva L.M., Pezarat-Correia P.C. (2018). Effects of Dental Occlusion on Body Sway, Upper Body Muscle Activity and Shooting Performance in Pistol Shooters. Appl. Bionics Biomech..

[60] Ekuni D., Takeuchi N., Furuta M., Tomofuji T., Morita M. (2011). Relationship between Malocclusion and Heart Rate Variability Indices in Young Adults: A Pilot Study. Methods Inf. Med..

[61] Maixner W., Greenspan J.D., Dubner R., Bair E., Mulkey F., Miller V., Knott C., Slade G.D., Ohrbach R., Diatchenko L. (2011). Potential Autonomic Risk Factors for Chronic TMD: Descriptive Data and Empirically Identified Domains from the OPPERA Case-Control Study. J. Pain.

[62] Santana M.D.R., de Souza A.C.A., de Abreu L.C., Valenti V.E. (2013). Association between Oral Variables and Heart Rate Variability. Int. Arch. Med..

[63] Roi G.S., Sisca G., Perondi F., Diamante A., Nanni G. (2004). Post Competition Blood Lactate Accumulation. J. Sport. Sci..

[64] Aslan A., Acikada C., Güvenç A., Gören H., Hazir T., Özkara A. (2012). Metabolic Demands of Match Performance in Young Soccer Players. J. Sport. Sci. Med..

[65] Svensson M., Drust B. (2005). Testing Soccer Players. J. Sport. Sci..

[66] De Souza J.J., Leite J.S., Bahls R., Grande R.S., Santos F.A. (2021). Clinical and Behavioral Conditions in Oral Health of Volleyball and Soccer Athletes: A Cross-Sectional Study. Braz. J. Oral Sci..

[67] Michalakis K.X., Kamalakidis S.N., Pissiotis A.L., Hirayama H. (2019). The Effect of Clenching and Occlusal Instability on Body Weight Distribution, Assessed by a Postural Platform. Biomed. Res. Int..

[68] Julià-Sánchez S., Álvarez-Herms J., Cirer-Sastre R., Corbi F., Burtscher M. (2019). The Influence of Dental Occlusion on Dynamic Balance and Muscular Tone. Front. Physiol..

[69] Ackerman J.L. (1974). Orthodontics: Art, Science, or Trans-Science?. Angle Orthod..

[70] Fleming P.S., Seehra J., Polychronopoulou A., Fedorowicz Z., Pandis N. (2013). Cochrane and Non-Cochrane Systematic Reviews in Leading Orthodontic Journals: A Quality Paradigm?. Eur. J. Orthod..

[71] Klages U., Bruckner A., Zentner A. (2004). Dental Aesthetics, Self-Awareness, and Oral Health-Related Quality of Life in Young Adults. Eur. J. Orthod..

[72] Helm S., Petersen P.E., Kreiborg S., Solow B. (1986). Effect of Separate Malocclusion Traits on Concern for Dental Appearance. Community Dent. Oral Epidemiol..

[73] Mahmood T.M.A., Kareem F.A. (2013). Psychological Impact of Dental Aesthetics for Kurdish Young Adults Seeking Orthodontic Treatment. Int. J. Health Rehabil. Sci. IJHRS.

[74] Hamdan A.M., Al-Omari I.K., Al-Bitar Z.B. (2007). Ranking Dental Aesthetics and Thresholds of Treatment Need: A Comparison between Patients, Parents, and Dentists. Eur. J. Orthod..

[75] Aksakalli S., Temucin F., Pamukcu A., Ezirganlı S., Kazancioglu H.O., Malkoc M.A. (2015). Effectiveness of Two Different Splints to Treat Temporomandibular Disorders. J. Orofac. Orthop..

[76] Zonnenberg A.J.J., Mulder J. (2014). The Efficacy of a Specific Stabilization Splint. Cranio.

[77] Wänman A., Marklund S. (2020). Treatment Outcome of Supervised Exercise, Home Exercise and Bite Splint Therapy, Respectively, in Patients with Symptomatic Disc Displacement with Reduction: A Randomised Clinical Trial. J. Oral Rehabil..

[78] Conti P.C.R., da Mota Corrêa A.S., Lauris J.R.P., Stuginski-Barbosa J. (2015). Management of Painful Temporomandibular Joint Clicking with Different Intraoral Devices and Counseling: A Controlled Study. J. Appl. Oral. Sci..

[79] Zou J., Meng M., Law C.S., Rao Y., Zhou X. (2018). Common Dental Diseases in Children and Malocclusion. Int. J. Oral Sci..

[80] Alqahtan I.M., Azizkhan R.A., Alyawer L.T., Alanazi S.S., Ahmed R., Alhazmi L.S., Bsher F.F., Zahran L.M., Aljahdali R.A., Alqwizany R.R. (2020). An Overview of Diagnosis and Management of Malocclusion: Literature Review. Ann. Dent. Spec..

[81] Zebrick B., Teeramongkolgul T., Nicot R., Horton M.J., Raoul G., Ferri J., Vieira A.R., Sciote J.J. (2014). ACTN3 R577X Genotypes Associate with Class II and Deepbite Malocclusions. Am. J. Orthod. Dentofac. Orthop..

[82] North K.N., Beggs A.H. (1996). Deficiency of a Skeletal Muscle Isoform of Alpha-Actinin (Alpha-Actinin-3) in Merosin-Positive Congenital Muscular Dystrophy. Neuromuscul. Disord..

[83] Zouhal H., Coso J.D., Jayavel A., Tourny C., Ravé G., Jebabli N., Clark C.C.T., Barthélémy B., Hackney A.C., Abderrahman A.B. (2021). Association between ACTN3 R577X Genotype and Risk of Non-Contact Injury in Trained Athletes: A Systematic Review. J. Sport Health Sci..

[84] Houweling P.J., Papadimitriou I.D., Seto J.T., Pérez L.M., Coso J.D., North K.N., Lucia A., Eynon N. (2018). Is Evolutionary Loss Our Gain? The Role of ACTN3 p.Arg577Ter (R577X) Genotype in Athletic Performance, Ageing, and Disease. Hum. Mutat..

[85] Hogarth M.W., Garton F.C., Houweling P.J., Tukiainen T., Lek M., Macarthur D.G., Seto J.T., Quinlan K.G.R., Yang N., Head S.I. (2016). Analysis of the ACTN3 Heterozygous Genotype Suggests That α-Actinin-3 Controls Sarcomeric Composition and Muscle Function in a Dose-Dependent Fashion. Hum. Mol. Genet..

[86] Seto J.T., Lek M., Quinlan K.G.R., Houweling P.J., Zheng X.F., Garton F., MacArthur D.G., Raftery J.M., Garvey S.M., Hauser M.A. (2011). Deficiency of α-Actinin-3 Is Associated with Increased Susceptibility to Contraction-Induced Damage and Skeletal Muscle Remodeling. Hum. Mol. Genet..

[87] Yang N., Schindeler A., McDonald M.M., Seto J.T., Houweling P.J., Lek M., Hogarth M., Morse A.R., Raftery J.M., Balasuriya D. (2011). α-Actinin-3 Deficiency Is Associated with Reduced Bone Mass in Human and Mouse. Bone.

[88] Cunha A., Nelson-Filho P., Marañón-Vásquez G.A., de Carvalho Ramos A.G., Dantas B., Sebastiani A.M., Silvério F., Omori M.A., Rodrigues A.S., Teixeira E.C. (2019). Genetic Variants in ACTN3 and MYO1H Are Associated with Sagittal and Vertical Craniofacial Skeletal Patterns. Arch. Oral Biol..

[89] Bichara L.M., Aragón M.L., Brandão G.A., Normando D. (2016). Factors influencing orthodontic treatment time for non-surgical Class III malocclusion. J. Appl. Oral Sci..

